# MicroRNA-181a Inhibits Activated B-Cell-Like Diffuse Large B-Cell Lymphoma Progression by Repressing *CARD11*

**DOI:** 10.1155/2019/9832956

**Published:** 2019-09-24

**Authors:** Danxia Zhu, Cheng Fang, Wenting He, Chen Wu, Xiaodong Li, Jun Wu

**Affiliations:** Department of Oncology, The Third Affiliated Hospital of Soochow University, Changzhou, China

## Abstract

We investigated the role of miR-181a in diffuse large B-cell lymphoma (DLBCL) and its potential target genes. miR-181a levels were lower in activated B-cell- (ABC-) like DLBCL cells than that in germinal center B-cell- (GCB-) like DLBCL cells. Overexpression of miR-181a in ABC-like DLBCL cell lines (OCI-LY10 and U2932) resulted in G0/G1 cell cycle arrest, increased apoptosis, and decreased invasiveness. miRNA target prediction programs (miRanda, TargetScan, and miRDB) identified caspase recruitment domain-containing protein 11 (*CARD11*) as a putative miR-181a target. *CARD11* mRNA and protein levels were higher in the ABC-like DLBCL than that in GCB-like DLBCL. Moreover, *CARD11* mRNA and protein levels were downregulated in the OCI-LY10 and U2932 cell lines overexpressing miR-181a. Dual luciferase reporter assays confirmed the miR-181a binding site in the CARD11 3′UTR region. OCI-LY10 and U2932 cells transfected with a *CARD11* expression vector encoding miR-181a with a mutated binding site showed higher *CARD11* protein levels, cell viability, G2/M phase cells, and invasiveness compared to those transfected with a wild-type *CARD11* expression vector. Nude mice xenografted with OCI-LY10 cells with overexpressed wild-type miR-181a generated smaller tumors compared to those with overexpressed mutated binding site of *CARD11* 3′UTR and miR-181a. These results indicate that miR-181a inhibits ABC-like DLBCL by repressing *CARD11*.

## 1. Introduction

Diffuse large B-cell lymphoma (DLBCL) is a malignant lymphoma that is highly heterogeneous. Despite greater understanding of the DLBCL pathological subtypes and effectiveness of rituximab-based chemoimmunotherapy, 35%–40% of patients show diminished treatment efficacy with rapid emergence of drug resistance [1, 2]. Therefore, there is an urgent need for in-depth understanding of DLBCL pathogenesis and the mechanisms that lead to drug resistance in order to improve survival rates.

MicroRNAs (miRNAs) are involved in tumorigenesis of many malignant tumors including DLBCL. They regulate cell differentiation, proliferation, apoptosis, and other basic cellular processes and play an important role in tumor diagnosis and prognosis [3, 4]. miR-181a/b plays an important role in the early development of B-lymphocytes, positive selection of T-cells and development of NK cells. The miR-181 family is involved in immune regulation, angiogenesis, tumor resistance to chemotherapy, and prognosis evaluation [5–8]. miR-181a/b suppresses the malignant transformation of B-lymphocytes by inhibiting cytidine deaminase (AID), BCL-6, FOXP-1, and other key genes that are involved in malignant transformation and differentiation of B-cells [5, 9, 10].

In our previous study, we analyzed 866 human miRNAs in six Chinese patients with chronic lymphocytic leukemia (CLL) and peripheral B-cells from pooled 30 healthy donors [11]. We demonstrated that miRs-126, -572, -494, -923, -638, -130a, -181a, and -181b were downregulated and miRs-29a, -660, -20a, -106b, -142-5p, -101, -30b, -34a, -let-7f, -21, and -155 were upregulated in CLL. Moreover, low miR-181a and miR-181b expression were associated with shorter overall survival and treatment-free survival in CLL patients [11]. In this study, we investigated the role of miR-181a/b in DLBCL and its target genes that are relevant for tumor growth and progression.

## 2. Materials and Methods

### 2.1. Cell Culture

SU-DHL4, U2932, OCI-Ly19, and OCI-LY10 cells were purchased from the Type Culture Collection of the Chinese Academy of Sciences (Shanghai, China). SU-DHL4 and U2932 cells were grown in RPMI 1640 with 10% FBS; OCI-Ly19 and OCI-LY10 were grown in IMDM with 20% human serum at 37°C in a humidified incubator with 5% CO_2_. The cell lines were authenticated by single nucleotide polymorphism profiling (fingerprinting).

### 2.2. Stably Transfected ABC-DLBCL Cell Lines

For the miR-181a or *CARD11* overexpression experiments, OCI-LY10 and U2932 cells were transduced with a lentiviral vector (Clontech) that constitutively expressed miR-181a precursor or *CARD11* driven by a CMV (cytomegalovirus) promoter. The lentiviral vector system included three plasmids, namely, the pLVX-CMV-GFP-puro vector, psPAX, and pMD2G. The resulting lentiviral vector with miR-181a precursor or *CARD11* was confirmed by PCR and sequencing.

To generate lentivirus, the HEK293 cell line was grown in the DMEM medium (ThermoFisher) supplemented with 10% fetal calf serum (ThermoFisher) and transfected with 1.8 ml DNA solution containing 10 *μ*g Lv-miR-181a or Lv-*CARD11*, 10 *μ*g psPAX, and 10 *μ*g pMD2G. All virus stocks were produced by calcium phosphate-mediated transfection. Cell supernatants containing viral particles were collected at 48 h after transfection and were filtered using the 0.45-*μ*m Steriflip vacuum filtration system (Millipore) and concentrated by ultracentrifugation at 25,000 rpm at 4°C. The titer of the virus was determined by the GFP expression. The day before infection, the OCI-LY10 and U2932 cells were seeded in dishes at a concentration of 10^6^/ml. On the day of infection, the OCI-LY10 and U2932 cells were infected by the packaged lentivirus with a MOI of 100, whereas uninfected OCI-LY10 and U2932 cells were cultured in parallel. After 72 h, the cells were selected by growing in the selection medium containing puromycin (300 mg/ml) for 2 weeks.

### 2.3. Quantitative Real-Time PCR (qRT-PCR)

Total RNA was extracted from cell lines using the miRNeasy isolation kit (Qiagen) and was quantified by NanoDrop 2000 (Thermo Fisher Scientific, MA, US). For miRNA quantification, 5 *μ*l of total RNA (1 ng/*μ*l) was mixed with 10 *μ*l TaqMan MicroRNA Reverse Transcription Kit reagent with specific miRNA primers and reverse transcribed at 16°C for 30 minutes, 42°C for 30 minutes, and 95°C for 5 minutes.

To quantify *CARD11* mRNA expression, 1 *μ*g RNA was reverse transcribed with the High-Capacity cDNA Reverse Transcription Kit (Applied Biosystems) according to the manufacturer's protocol with a minor modification (1 U/ul RNase inhibitor (Applied Biosystems) was added). The reaction was carried out at 25°C for 10 minutes and 37°C for 60 minutes. Real-time PCR was performed as follows: 94°C for 5 min and 40 cycles at 94°C for 30 s, 55°C for 30 s, and 72°C for 30 s in the ABI PRISM 7900HT Sequence Detection System Instrument (Applied Biosystems). Glyceraldehyde 3-phosphate dehydrogenase (GAPDH) and U6 were used as internal controls of *CARD11* and miR-181a expression, respectively. Data were analyzed by the comparison Ct (2^−ΔΔCt^) method and expressed as fold change relative to GAPDH or U6. The primer sequences are shown in Table 1. Each sample was analyzed in triplicate.

### 2.4. CCK-8 Cell Viability Assay

The cell viability was monitored by the Cell Counting Kit-8 (CCK8) assay kit (Dojindo Molecular Technologies, Kumamoto, Japan) according to the manufacturer's protocol. In brief, cells were seeded into 96-well plates at a density of 1 × 10^4^ cells/well. Detection was done in 6 wells per group, and blank controls were also detected. At 0, 24 h, 48 h, 72 h, and 96 h, 10 *μ*l of CCK-8 solution was added, followed by incubation for 3 h at 37°C. The optical density (OD) was measured at 450 nm to reflect the cell viability. The experiments were repeated thrice.

### 2.5. Transwell Matrigel Cell Invasion Assay

Transwell invasion assay was performed in 24-well BD Matrigel invasion chambers (BD Biosciences) according to the manufacturer's instructions. A total of 5 × 10^4^ cells per well were seeded in the upper well with the DMEM medium without serum, whereas the lower chamber was filled with the DMEM medium containing 10% FBS. After 24 hours of incubation, the noninvading cells in the top well were removed with a cotton swab and the cells in the bottom well were fixed with 3% paraformaldehyde. Subsequently, the cells were stained with 0.1% crystal violet, extracted with 33% acetic acid and quantified in a standard microplate reader at 570 nm.

### 2.6. Western Blot Analysis

Xenograft tumor tissue samples (200–300 mg) and cell pellets were homogenized in 10 volumes of lysis buffer (20 mM Tris-HCl (pH, 7.4), 150 mM NaCl, 2.5 mM EDTA, 50 mM NaF, 0.1 mM Na_4_P_2_O_7_, 1 mM Na_3_VO_4_, 1 mM PMSF, 1 mM DTT, 0.02% (v/v) protease cocktail (Sigma-Aldrich, Missouri, USA), 1% (v/v) Triton X-100, and 10% (v/v) glycerol). The homogenates were centrifuged twice at 20,000 g at 4°C for 15 min, and the protein supernatants were quantified by the BCA method. Equal amounts of proteins were separated by SDS-PAGE. The separated proteins were transferred to a PVDF membrane (Bio-Rad, California, USA). The blots were then incubated with anti-*CARD11* (1 : 2000 dilution, Ab91463, Abcam, USA) and anti-glyceraldehyde-3-phosphate dehydrogenase (GAPDH, 1 : 5000 dilution, Ab8245, Abcam, USA) antibodies overnight at 4°C. Then, after washing with 1X TBST buffer twice, the blots were incubated with horseradish peroxidase conjugated goat anti-mouse secondary antibody (1 : 8000, Sigma Aldrich) at room temperature for 1 h. Then, the blots were developed with ECL enhanced chemiluminescent detection kit (Amersham, London, UK) and exposed to X-ray film. The protein bands were quantified by densitometry with Bio-Rad Gel Doc 2000 system (Bio-Rad, California, USA).

### 2.7. Flow Cytometry Analysis of Cell Cycle and Apoptosis

For cell cycle analysis, single cell suspensions were fixed with 70% ethanol for 30 min at 4°C followed by RNA digestion with RNAase (0.5 mg/ml). Then, the permeabilized cells were labeled with propidium iodide (5 mg/ml; Sigma-Aldrich, MO, USA). Subsequently, DNA content was assessed by using an Epics xL flow cytometer (Beckman Coulter, U. K.). For the cell apoptosis assay, the cells were stained with APC conjugated anti-AnnexinV antibody and propidium iodide (PI) according to the manufacturer's protocol manufacturer (BioVision Inc., Milpitas, CA, USA). The percentage of AnnexinV^+^ PI^+^ cells were determined by using an Epics xL flow cytometer (Beckman Coulter, UK).

### 2.8. Luciferase Reporter Assay

The putative miR-181a binding sequence in the 3′-UTR (1–503 bp) of h*CARD11* (5′-agagcCAGAGCAGCAGUUGAAUGUa-3′) or a mutated variant (5′-agagc CAGAGCAGCAGGGCGGACUa-3′) was cloned into the psiCheck2 firefly-luciferase vector (Promega). The firefly luciferase construct was cotransfected with a control Renilla luciferase vector into OCI-LY10 and U2932 cells in the presence of miR-181a or control lentiviral vectors (Lv-miR-181a or Lv-control). A dual luciferase assay (Promega) was performed 48 hours after transfection. Renilla luciferase activity was normalized to Firefly luciferase activity. The experiments were repeated thrice.

### 2.9. Tumor Growth in the Xenograft Mouse Model

The animal experiments were performed according to the recommendations in the Guide for the Care and Use of Laboratory Animals of the Third Affiliated Hospital of Soochow University, and the animal studies were approved by the ethics committee of the Third Affiliated Hospital of Soochow University. Six SCID mice per group were injected subcutaneously with 1 × 10^7^ OCI-LY10 cells (200 *μ*l PBS) that were transfected with (1) vector control, (2) lentiviral vectors with miR-181a plus wild-type *CARD11*, and (3) lentiviral vectors with miR-181a plus mutant *CARD11*. The xenograft tumors were measured every 5 days to determine tumor volume as (1/2) × length × width^2^. The mice were sacrificed on the 30^th^ day, and tumors were harvested and weighed to determine the tumor weights.

### 2.10. Statistical Analysis

Data were analyzed using SPSS 16.0 software package and expressed as mean ± SEM. Statistical significance was determined by ANOVA or repeated ANOVA for multiple comparisons or repeated measurements. Significant differences between two mean values were estimated using Student's *t*-test. *P* < 0.05 was considered statistically significant.

## 3. Results

### 3.1. Decreased miR-181a Expression in ABC-DLBCL Cell Lines

**Figure 1:**
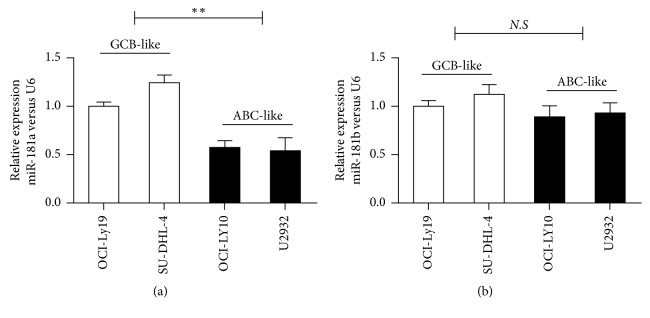
Relative miR-181a/b expression in ABC- and GCB-DLBCL cell lines. Histograms show relative miR-181a (a) and miR-181b (b) expression in GCB-like (OCI-Ly19 and SU-DHL-4) and ABC-like (OCI-LY10 and U2932) DLBCL cell lines. Note*:* two independent experiments were conducted in triplicates per cell line; values represent mean ± SEM; statistical significance was determined by the unpaired *t*-test, two-sided; ^*∗∗*^*P* < 0.01 when compared with GCB-like DLBCL cell lines.

As shown in Figure 1, qRT-PCR analysis showed decreased miR-181a levels in the ABC-like DLBCLs (OCI-LY10 and U2932) than that in GCB-like DLBCLs (OCI-Ly19 and SU-DHL-4) (Figure 1). However, miR-181b levels were similar between GCB- and ABC-like DLBCL cell lines. This suggested that miR-181a expression may be associated with the differential development of the different DLBCL subgroups. We chose OCI-LY10 and U2932 cells for further studies.

### 3.2. Effects of miR-181a Overexpression in OCI-LY10 and U2932 Cells on Cell Cycle, Viability, and Invasion

**Figure 2:**
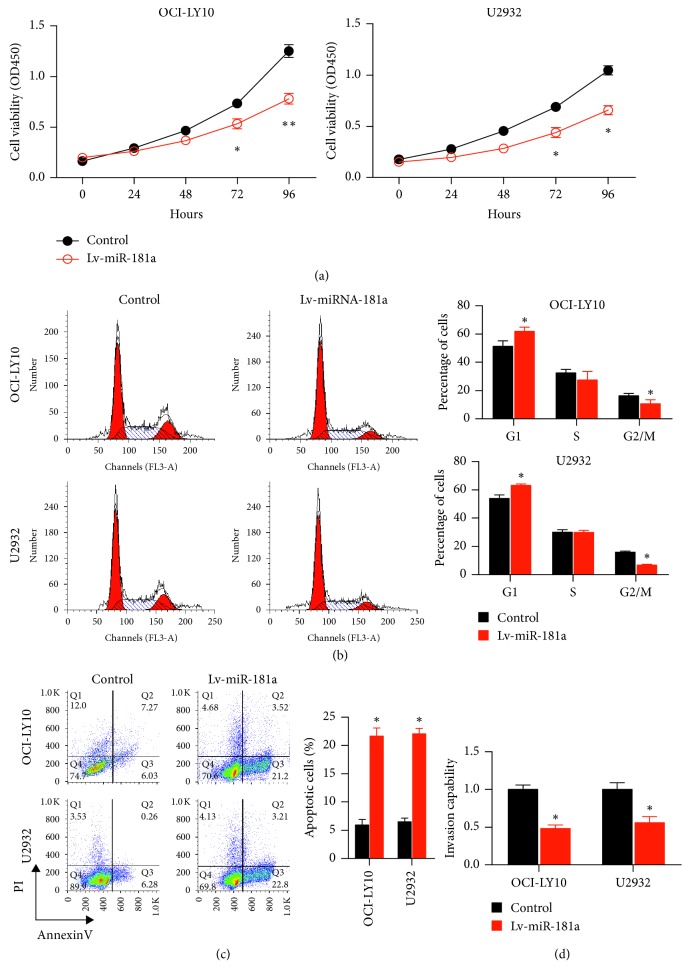
Effects of miR-181a overexpression on cell cycle, proliferation, viability, and invasiveness of ABC-DLBCL cell lines. (a) CCK-8 assay shows decreased proliferation in OCI-LY10 (left panel) and U2932 (right panel) cell lines stably expressing miR-181a than that in controls. (b) FACS plots (left panel) depicting PI stained control and miR-181a overexpressing OCI-LY10 (top) and U2932 (below) cell lines demonstrating their cell cycle (G0/G1, S, and G2/M) distribution. Right panel shows quantification demonstrating increased percent G0/G1 phase cells and reduced G2/M phase cells in miR-181a overexpressing OCI-LY10 and U2932 cell lines. (c) Flow cytometry apoptosis analysis showing AnnexinV versus PI plots of control and miR-181a overexpressing OCI-LY10 (top) and U2932 (bottom) cells. Right panel shows histograms showing percent apoptotic cells (AnnexinV^+^ PI^+^) in control (black) and miR-181a overexpressing (red) OCI-LY10 and U2932 cells. As shown, miR-181a overexpression increased apoptosis in both DLBCL cell lines. (d) Transwell matrigel analysis showing the relative number of invading cells in control (black) and miR-181a overexpressing (red) OCI-LY10 and U2932 cells. The controls were set to 1.0. As shown, miR-181a overexpression decreased invasiveness of both DLBCL cell lines. Note: results are expressed as mean ± SEM of three independent experiments; ^*∗*^*P* < 0.05 and ^*∗∗*^*P* < 0.01 versus control group.

The CCK-8 assay showed decreased proliferation of OCI-LY10 and U2932 cells transfected with miR-181a overexpressing lentiviral vector than that in controls (*P* < 0.05; Figure 2(a)). The miR-181a overexpressing OCI-LY10 and U2932 cells showed higher G0/G1 and lower G2/M phase cells than that in the controls, suggesting G0/G1 cell cycle arrest (Figure 2(b)). FACS analysis with AnnexinV/PI staining showed increased apoptosis in miR-181a overexpressing OCI-LY10 and U2932 cells than that in the controls (Figure 2(c)). Transwell matrigel assays showed that miR-181a overexpression in OCI-LY10 and U2932 cells led to less invasiveness compared to the controls (Figure 2(d)). These data demonstrated that miR-181a acted as a tumor suppressor in ABC-DLBCL cells.

### 3.3. *CARD11* Is a miR-181a Target Gene in ABC-DLBCL

**Figure 3:**
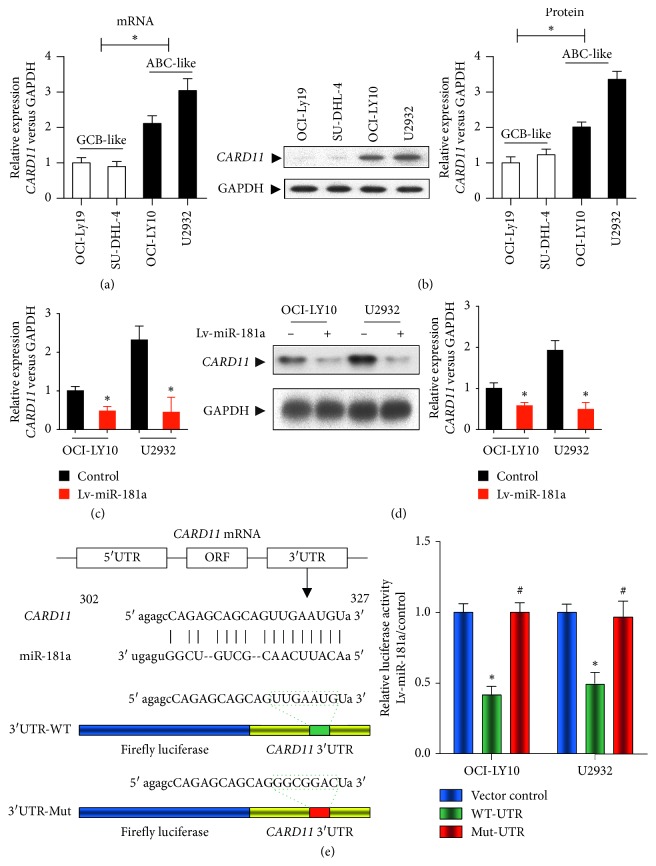
miR-181a suppresses *CARD11* expression in ABC-DLBCLs. (a) QRT-PCR analysis shows relative expression of *CARD11* in GCB-like (OCI-Ly19 and SU-DHL-4) and ABC-like (OCI-LY10 and U2932) DLBCL cell lines. Expression was normalized to U6 levels. (b) Representative western blot (left panel) shows *CARD11* protein expression in GCB-like (OCI-Ly19 and SU-DHL-4) and ABC-like (OCI-LY10 and U2932) DLBCL cell lines. Right panel shows quantification of *CARD11* protein normalized against GAPDH. (c) QRT-PCR analysis shows relative expression of miR-181a (versus GAPDH) in stably transfected miR-181a overexpressing OCI-LY10 and U2932 cells relative to controls transfected with empty vector. (d) Representative western blot shows decreased *CARD11* protein expression in miR-181a overexpressing OCI-LY10 and U2932 cell lines than that in controls. (e) Left panel shows schematics of (top) *CARD11* mRNA with putative miR-181a binding site in its 3′-untranslated region (3′-UTR) and (b) luciferase reporter constructs with *CARD11* mRNA sequences with wild-type or mutated 3′-UTR miR-181a binding sites. Right panel shows relative luciferase activity in OCI-LY10 and U2932 cell lines cells transfected with vector control (blue), *CARD11* mRNA with wild-type 3′UTR containing the miR-181a binding site (green) and *CARD11* mRNA with 3′UTR containing mutated miR-181a binding site (red). Luciferase activity was measured 48 h after cotransfection. Note: results are expressed as mean ± SEM of three independent experiments; ^*∗*^*P* < 0.05 versus vector control group; ^#^*P* < 0.05 versus wt-UTR group.

We analyzed downstream targets of miR-181a with three miRNA target prediction programs, miRanda, TargetScan, and miRDB (Supplementary [Supplementary-material supplementary-material-1]), that identified *CARD11* as a putative target (Figure 3(e)). High *CARD11* mRNA and protein levels in the ABC-DLBCL cell lines suggested negative correlation between miR-181a and *CARD11* in DLBCL (Figures 3(a) and 3(b)). This was further corroborated by decreased *CARD11* expression in miR-181a overexpressing OCI-LY10 and U2932 cells compared to the controls (Figures 3(c) and 3(d)). Then, fragments of the 3′-UTR of *CARD11* were cloned with the wild-type or mutated miR-181a binding site into a firefly luciferase reporter vector (Figure 3(e)). Luciferase activity was reduced by approximately 55% in OCI-LY10 and 51% in U2932 cells comparing to the controls, thereby confirming that *CARD11* is a direct target of miR-181a in ABC-DLBCL cells (Figure 3(e)).

### 3.4. *CARD11* Mediates miR-181a Effects on ABC-DLBCL *In Vitro*

**Figure 4:**
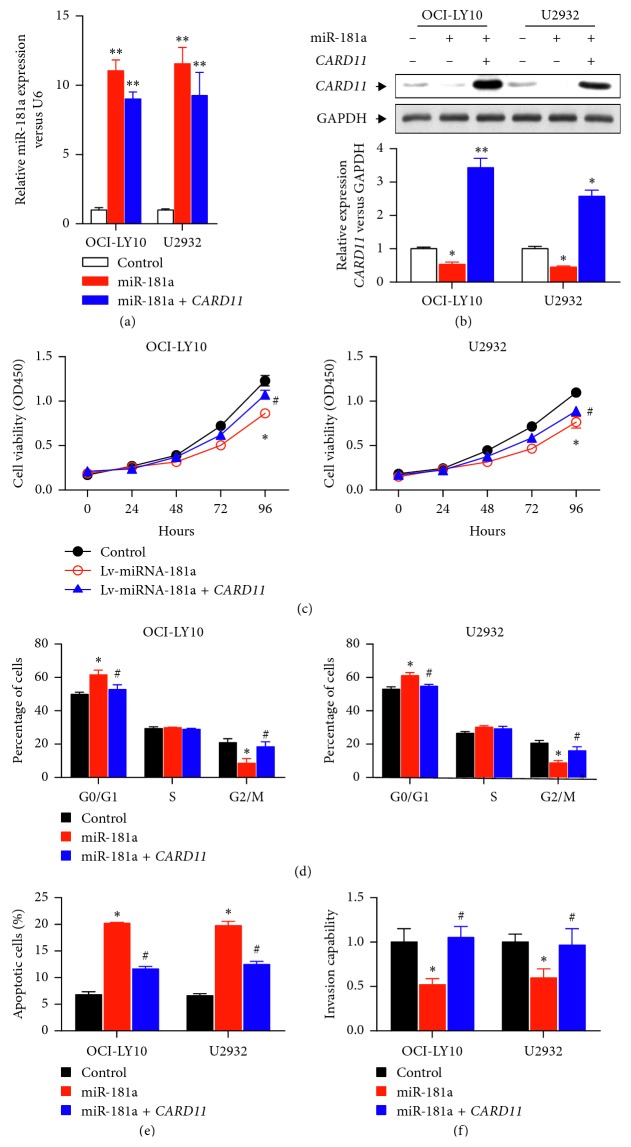
Effects of miR-181a and *CARD11* overexpression on ABC-DLBCL cells *in vitro*. (a) Relative miR-181a levels in OCI-LY10 and U2932 cells transfected with vector-only (white bar) or *CARD11*-overexpressing plasmids with wild-type (red) or mutated (blue) miR-181a binding site. (b) Western blot analysis of *CARD11* levels in in OCI-LY10 and U2932 cells transfected with vector-only (white bar) or *CARD11*-overexpressing plasmids with wild-type (red) or mutated (blue) miR-181a binding site. (c) CCK-8 assay showing cell proliferation status of OCI-LY10 (left panel) and U2932 (right panel) cells transfected with vector-only (black line) or *CARD11*-overexpressing plasmids with wild-type (red line) or mutated (blue line) miR-181a binding site. (d) Cell cycle analysis showing percent G0/G1, S, and G2/M phase cells in OCI-LY10 (left panel) and U2932 (right panel) cells transfected with vector-only (black bar) or *CARD11*-overexpressing plasmids with wild-type (red bar) or mutated (blue line) miR-181a binding site as analyzed by flow cytometry. (e) Flow cytometry analysis of percent apoptotic cells (AnnexinV^+^PI^+^) in OCI-LY10 (left) and U2932 (right) cells transfected with vector-only (black bar) or *CARD11*-overexpressing plasmids with wild-type (red bar) or mutated (blue bar) miR-181a binding site. (f) Transwell matrigel assay showing invasiveness of OCI-LY10 (left) and U2932 (right) cells transfected with vector-only (black bar) or *CARD11*-overexpressing plasmids with wild-type (red bar) or mutated (blue bar) miR-181a binding site. Note: ^*∗*^*P* < 0.05 and ^*∗∗*^*P* < 0.01 versus control group; ^#^*P* < 0.05 versus Lv-miR-181a group; results are expressed as mean ± SEM of three independent experiments.

Cotransfection of miR-181a and wild-type *CARD11* overexpression plasmids in OCI-LY10 and U2932 cells decreased *CARD11* protein levels (Figure 4(a)). However, miR-181a overexpression did not affect *CARD11* protein levels in cells transfected with *CARD11* overexpression plasmid with mutated miR-181a binding sites (Figure 4(b)). The CCK-8 assay showed that the cells with mutant *CARD11* overexpression plasmid promoted cell growth despite increased miR-181a levels (Figure 4(c)). Cell cycle analysis demonstrated increased G2/M and decreased G0/G1 phase cells in OCI-LY10 and U2932 cells with mutant *CARD11* overexpression plasmid (Figure 4(d)). Furthermore, OCI-LY10 and U2932 cells with mutant *CARD11* overexpression plasmid showed reduced apoptosis despite miR-181a overexpression compared to OCI-LY10 and U2932 cells with wild-type *CARD11* overexpressing plasmid (Figure 4(e)). Moreover, transwell matrigel assays demonstrated increased invasiveness of OCI-LY10 and U2932 cells transfected with mutant *CARD11* overexpression plasmid compared to those transfected with the wild-type *CARD11* OE plasmid in addition to miR-181a overexpression (Figure 4(f)). These data demonstrated that miR-181a mediates proliferation, invasion, and antiapoptosis by transcriptionally modulating *CARD11* expression.

### 3.5. miR-181a Inhibits ABC-DLBCL Tumorigenesis by Suppressing *CARD11*

**Figure 5:**
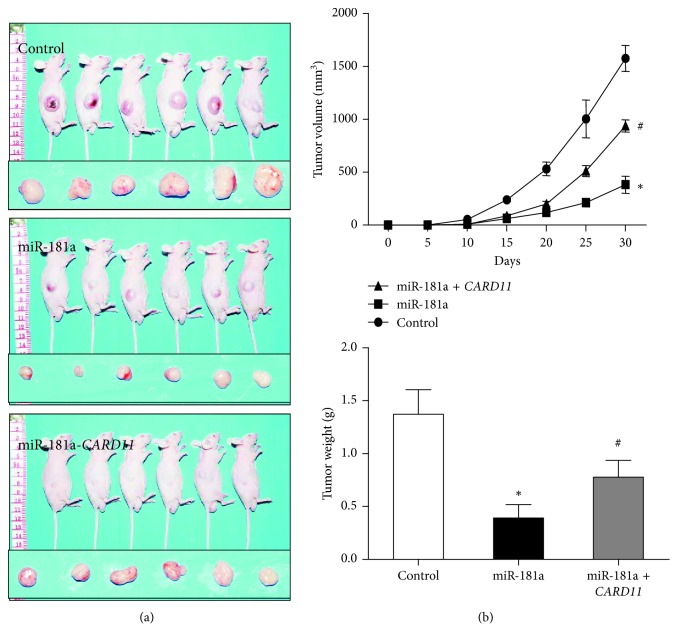
miR-181a regulates xenograft tumor growth of ABC-DLBCL *in vivo* by repressing *CARD11*. (a) Representative images of mice xenografted with OCI-LY10 cells transfected with vector control (top) or *CARD11*-overexpressing plasmids with wild-type (miR-181a) or mutated (miR-181a-*CARD11*) miR-181a binding site. Also shown are the harvested tumors at the end of the experiment (30 days). (b) The growth of tumor as analyzed by tumor volume between days 0–30 (top panel), and tumor weights on day 30 (bottom panel) are shown. Note: tumor volume of xenografts was measured with calipers every 5 days for 30 days. ^*∗*^*P* < 0.05 versus control group; ^#^*P* < 0.05 versus Lv-miR-181a group.

To further confirm if miR-181a inhibits ABC-DLBCL tumorigenesis *in vivo*, we used the SCID mice xenograft model with OCI-LY10 cells transfected with (1) control, (2) miR-181 and *CARD11* overexpression vectors with wild-type miR-181a binding sites, and (3) miR-181 and *CARD11* overexpression vectors with mutant miR-181a binding sites. We observed that miR-181a overexpressing cells decreased tumor growth compared to the control cells, but OCI-LY10 cells expressing mutant *CARD11* overexpression plasmid without miR-181 binding sites showed increased tumor growth despite miR-181 overexpression (Figure 5). This demonstrated that miR-181a inhibited ABC-DLBCL tumorigenesis by suppressing *CARD11*.

## 4. Discussion

DLBCL is the most common type of lymphoma accounting for 30%∼40% of non-Hodgkin lymphoma with significant heterogeneity in its clinical manifestation, pathogenesis, and prognosis. Based on gene expression profiling and tumor cell origins, DLBCL is classified into germinal center B-cell (GCB) type and activated B-cell (ABC) type [12, 13]. The proportion of the ABC type is higher in Chinese patients than that in western countries and accounts for 70%–80% of DLBCL cases [14]. Chemoimmunotherapy combined with rituximab (R-CHOP) is the standard treatment for DLBCL, but the prognosis for ABC-type patients is worse than GBC-type ones [15, 16].

miRNAs are noncoding RNAs that are involved in various biological processes through posttranscriptional regulation. Aberrant miRNA expression mediates the tumor proliferation and tumor drug resistance by targeting cell cycle, apoptotic, and other signaling pathways [17, 18]. To date, there have been relatively few studies on the relationship between miRNA and prognosis, drug resistance, and proliferation in DLBCL. Alencar et al. showed that miR-181a was an independent prognostic factor in patients with DLBCL after standard R-CHOP chemotherapy; patients with low miR-181a expression had shortened progression-free survival (PFS), and high miR-181a expression inhibited FOXP-1 gene expression in the ABC-type of DLBCL [10]. de Yébenes et al. reported that miR-181b regulated the B-cell antibody-type conversion and recombination process and suppressed malignant transformation by inhibiting AID gene expression [9]. Zhang et al. showed that miR-181b was highly expressed in GCB-type B-cells compared to that in non-GCB-type cells (memory B-cells and plasma cells) [19]. This suggested that miR-181a/b was important for the B-cell differentiation process and was closely associated with the pathology of ABC/GCB-type DLBCL.

Most probably, miR-181a/b regulates genes related to the NF-*κ*B and p38/MAPK pathways because aberrant activation and high expression of the proteins related to these two pathways are closely associated with cell survival and resistance of DLBCL cells to immunochemotherapy; their inhibition significantly increases the therapeutic effects of immune chemotherapy [20–22].

We observed reduced miR-181a expression was in ABC-type DLBCL cells, whereas miR-181b expression was similar in ABC and GCB cell lines. *In vivo* and *in vitro* studies showed that exogenous overexpression of miR-181a significantly inhibited tumor growth by inhibiting *CARD11*. *CARD11* is the only lymphocyte-specific member of the membrane-associated guanylate kinase family that acts at the intersection of the BCR and TCR signaling pathways. *CARD11* was mutated and highly expressed in ABC DLBCL, which further activated the NF-*κ*B pathway leading to increased nuclear transcription of various target genes including BCL2, API2, and BCLX [23]. In a Middle Eastern population, high *CARD11* mRNA and protein expression were related to the prognosis of DLBCL [24]. Shigeo et al. found that the overexpression of *CARD11* and CARD9 was associated with gastric B lymphoma [25]. Ngo et al. performed loss of function screen of 2500 genes in DLBCL and found that *CARD11*, *MALT1*, and *BCL-10* were critical genes for tumor cell survival and proliferation with *CARD11* playing a key role in ABC-DLBCL [26]. Moreover, higher *CARD11* mRNA levels were observed in ABC-DLBCL biopsy specimens than that in GCB-DLBCL specimens. It was postulated that *CARD11* enabled cell survival through *BCL10*-dependent IKK activation in ABC-DLBCL. Furthermore, since *CARD11* is only expressed in lymphoid tissues, it is an attractive therapeutic target for ABC-DLBCL [27].

In conclusion, our study reveals that miR-181a suppresses ABC-DLBCL by targeting *CARD11* and has immense therapeutic potential.

## Figures and Tables

**Table 1:** Primer sequence used for quantitative real-time PCR.

Gene	Forward primer (5′-3′)	Reverse primer (5′-3′)
U6	GCTTCGGCAGCACATATACTAAAAT	CGCTTCACGAATTTGCGTGTCAT
*CARD11*	CGCACTTCCTGATGAACGAG	GTCCCGCTCTTCCTTCATCT
GAPDH	GGGAAACTGTGGCGTGAT	GAGTGGGTGTCGCTGTTGA

## Data Availability

The data used to support the findings of this study are available from the corresponding author upon request.

## Supplementary Materials

Supplementary Materials miRNA target prediction programs: miRDB, miRanda, and TargetScan.
