# Supplementary material for: MicroRNA-181a Inhibits Activated B-Cell-Like Diffuse Large B-Cell Lymphoma Progression by Repressing CARD11
Source: J Oncol. 2019 Sep 24;2019:9832956. doi: 10.1155/2019/9832956 (PMC6778910; doi:10.1155/2019/9832956)

The used softwares are as follows:

1) miRDB
[http://www.mirdb.org/miRDB](http://www.mirdb.org/miRDB/policy.html)


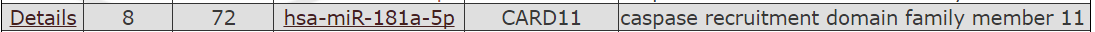


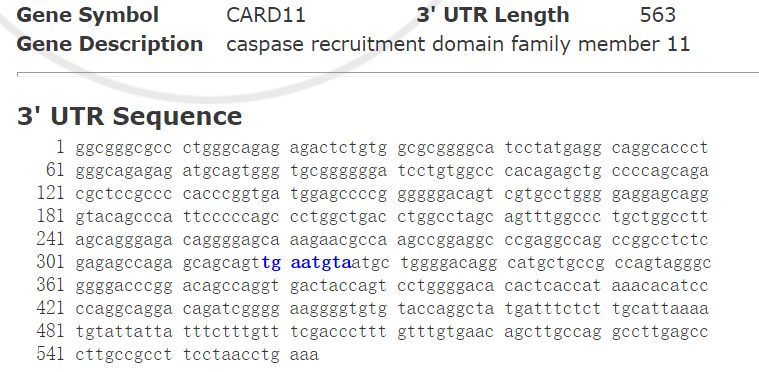


2) miRanda
[http://www.microrna.org/microrna](http://www.microrna.org/microrna/home.do)


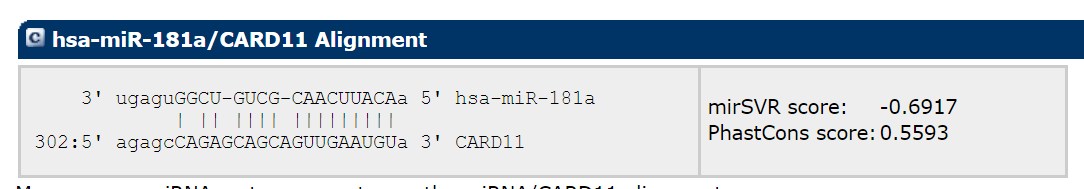
3) TargetScan
[http://www.targetscan.org](http://www.targetscan.org/vert_71/)


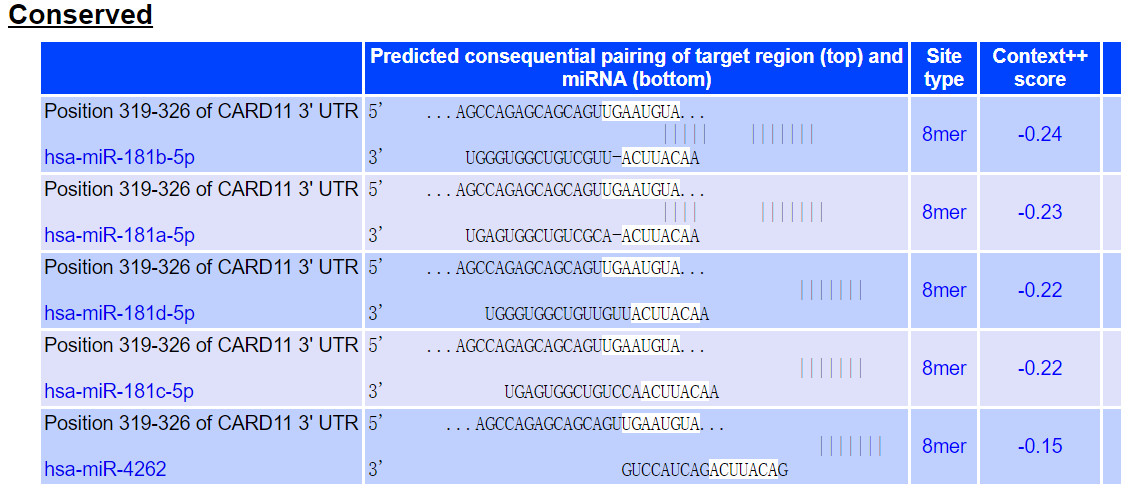

Supplement: Supplementary Materials — miRNA target prediction programs: miRDB, miRanda, and TargetScan. [file 9832956.f1.docx]
